# Best Graph Type to Compare Discrete Groups: Bar, Dot, and Tally

**DOI:** 10.3389/fpsyg.2021.775721

**Published:** 2021-12-24

**Authors:** Fang Zhao, Robert Gaschler

**Affiliations:** ^1^Research Cluster D2L2, FernUniversität in Hagen, Hagen, Germany; ^2^Faculty of Psychology, FernUniversität in Hagen, Hagen, Germany

**Keywords:** group comparison, graph comprehension, graph schema, mixing-costs paradigm, graph type

## Abstract

Different graph types might differ in group comparison due to differences in underlying graph schemas. Thus, this study examined whether graph schemas are based on perceptual features (i.e., each graph has a specific schema) or common invariant structures (i.e., graphs share several common schemas), and which graphic type (bar vs. dot vs. tally) is the best to compare discrete groups. Three experiments were conducted using the mixing-costs paradigm. Participants received graphs with quantities for three groups in randomized positions and were given the task of comparing two groups. The results suggested that graph schemas are based on a common invariant structure. Tally charts mixed either with bar graphs or with dot graphs showed mixing costs. Yet, bar and dot graphs showed no mixing costs when paired together. Tally charts were the more efficient format for group comparison compared to bar graphs. Moreover, processing time increased when the position difference of compared groups was increased.

## Introduction

**FIGURE 1:**
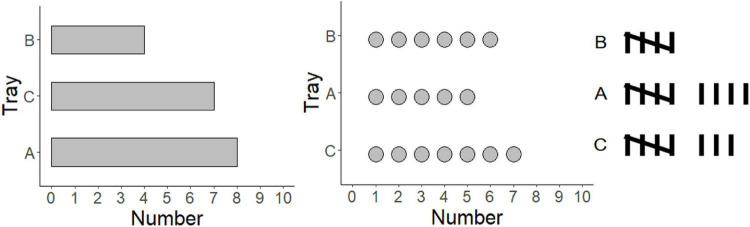
Examples of graph types used in Experiments 1–3: Bar graph, dot plot, and tally chart.

Data graphs are the ideal tools for comparing group differences. They are widely used in everyday life, such as in politics, sports, stock market reports, and scientific articles. Therefore, a crucial question is, how we extract specific information from graphs and which type of graph is the most suitable for group comparison. In theories of graph comprehension, activating the graph schema is the most important step (Simkin and Hastie, 1987; Pinker, 1990). There are, however, different hypotheses on whether the graph schema is based on specific perceptual features (i.e., each graph has a unique graph schema, Lohse, 1993) or common invariant structures (i.e., shared by several graphs, Ratwani and Trafton, 2008). Bar graphs have often been investigated, and it is suggested that bars are ideal for discrete comparisons (Pinker, 1990; Tversky et al., 2000; Shah and Freedman, 2009). However, other types of data graphs can also be used for group comparisons, such as dot plots or tally charts (Figure 1). This study, therefore, tested whether the graph schema is based on perceptual features or common invariant structures. In addition, it examined which graphic type (bar vs. dot vs. tally) is the most suitable for group comparison.

### Perceptual Features vs. Common Invariant Structure

Many theories of graph comprehension have been proposed to explain how we extract information from a graph (Pinker, 1990; Lohse, 1993; Shah and Carpenter, 1995; Peebles and Cheng, 2002, 2003). These theories take the perceptual process, short-term memory, and long-term memory into account (Shah and Carpenter, 1995; Shah and Hoeffner, 2002; Ratwani et al., 2008). First, the visual information in the data graph is decoded *via* pattern recognition processes. For instance, a bar graph is decoded as several closed boxes, labels, and x-y coordinates. Second, parts of a visual mental model about the decoded information become represented in the capacity-limited working memory. Third, the conceptual relations were retrieved from the long-term memory and the interpretive processes start. During the interpretive processes, the graph schema stored in the long-term memory is activated. A graph schema is a generic scaffold that directs readers to insert new information into a complex knowledge representation (Simkin and Hastie, 1987; Pinker, 1990). The graph schema describes relationships between ideas and fosters assumptions for the missing information (Simkin and Hastie, 1987). Integrating the perceived information with the graph schema is thus the most crucial step, as one activates the mental representation in the long-term memory and matches it to the early visual input. Fourth, the required information is determined. For instance, the required information in this study is the difference between group A and group B. Finally, the relevant information is located, and the answer is provided. For instance, the difference between groups A and B in the bar graph is 4 (Figure 1).

There are two main hypotheses regarding the structure of graph schemas. The hypothesis of *perceptual features* suggests that each type of graph is determined by a different schema, leading to the graphs being interpreted differently (Lohse, 1993). Each graph has its graphical pattern, which is the most distinguishable perceptual characteristic of a graph (Kosslyn, 1989). In other words, the perceptual feature view assumes that bar graphs have a unique graph schema and so do dot plots and tally charts. The hypothesis of the *invariant structure* suggests that the types of graphs are based on certain broad categories or shared common graph schemas (Peebles and Cheng, 2002, 2003; Ratwani and Trafton, 2008). Graph schemas are organized hierarchically with a general schema and graph-specific schemas. The general schema includes the common features of all graphs. The graph-specific schemas include the unique features of individual graphs. When a graph is perceived, one matches the graph with the corresponding graph schema, such as bar, line, and pie (Pinker, 1990). Then, the information is organized based on the activated graph schema. One uses the integrated information from the graph and the activated graph schema to derive new relations, such as comparing the values of two groups.

### Features of Bar, Dot, and Tally

Bar graphs consist of bars or closed containers in a Cartesian coordinate system with x- and y-axes (Tversky et al., 2000). The bars enclose one group and separate it from other groups. One axis represents the labels of the different groups. The other axis shows the values of the scale. Bar graphs are ideal for discrete comparisons (Cleveland, 1984; Cleveland and McGill, 1985; Carswell and Wickens, 1987; Shah et al., 1999). When asking participants to interpret and produce graphs, bar graphs are interpreted and used to present discrete comparisons, such as “male’s height is higher than that of female’s” (Zacks and Tversky, 1999). Bar graphs are commonly used in psychology (Witt, 2019). They appear to be less biased when describing the relationships between groups (Shah and Shellhammer, 1999), but when viewing the means of all groups, they appear lower in bar graphs than they do in dot plots (Godau et al., 2016).

The dot plot is a variant of bar graphs (Cleveland, 1984; Jacoby, 2006). Dots are shown instead of closed boxes (Figure 1). There can be dots with equal sizes (Sarkar and Rashid, 2021), or a larger dot at the highest value and many small dots in between (Cleveland and McGill, 1985). Alternatively, only one dot is shown at the highest value (Sönning, 2018). Dot plots can display frequency counts by showing the range of the values on one axis and labels of the values on the other axis. Dot plots can display a large amount of data, as it is not restricted to the width of the bars (Sönning, 2018). A dot plot with hundred values can be easily displayed on one page. As it uses the x-y coordinates, dot plots can provide relatively accurate perception (Cleveland, 1994).

Tally charts are comprised of every element of a dataset (Harris, 1999). The data can be shown either vertically or horizontally. Five tally marks are grouped into one unit by crossing the fifth mark over the first four (Figure 1). The tally charts grow longer with higher values. Tally charts are often used in school mathematic lessons to collect, count, and organize data (Davis, 2014). In this domain, they seem ideal to show frequency. For instance, a research project counting the number of vehicles in traffic (e.g., cars, vans, bicycles, and others) used tally charts, as children can easily add the numbers to each of the categories (Garcia Garcia and Cox, 2008). Tally charts have also been used by young children to record and count their favorite foods (Ashbrook, 2011). Kindergarten children can use tally charts to collect data based on their own interests and communicate with their peers (Curcio and Folkson, 1996). An interview study shows that pupils in Grades 2–5 preferred to use a tally chart to reorganize the data by frequency (Nisbet et al., 2003). However, it is not yet clear whether tally charts are more suitable for group comparisons compared to graphs using x-y coordinates. This study, therefore, aimed to scrutinize the ideal data graph (bar vs. dot vs. tally) to compare group differences and the underlying schema among data graphs.

### Mixing-Costs Paradigm

**FIGURE 2:**
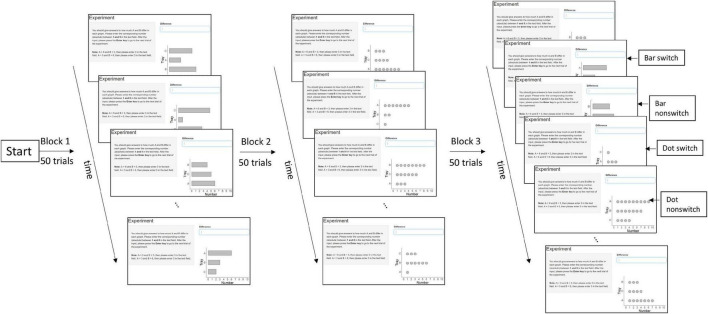
Design overview of Experiment 1 (bar vs. dot) in two pure blocks and one mixed block with switch and non-switch conditions. The order of the blocks is randomized. The design of Experiments 2 and 3 is similar to Experiment 1, except Experiment 2 used bar and tally and Experiment 3 used dot and tally.

The mixing-costs paradigm (Ratwani and Trafton, 2008) was used to distinguish the underlying graph schema in the sets of data graphs. This paradigm compares the reaction times (RTs) of the presented data graphs in pure blocks and a mixed block. Differences between the pure blocks and the mixed block can be attributed to different underlying graph schemas for each set of data graphs. For instance, to compare whether bar graph and dot plot share the same graph schema, the RTs in the pure block of bar graph and in the pure block of dot plot are compared with the RTs in the mixed block of bar graph and dot plot. If they share the same graph schema, there should be no time costs in the mixed block compared to the pure blocks, as no new graph schema needs to be activated. If they rely on different graph schemas, more time should be needed in the mixed block than pure blocks, because the appropriate graph schema needs to be retrieved. The mixed block has four conditions. Experiment 1 (bar vs. dot) has been taken as an example: *bar switch* refers to a dot plot that is preceded by a bar graph; *bar non-switch* means a bar graph that is preceded by a bar graph; *dot switch* refers to a bar graph that is preceded by a dot plot; and *dot non-switch* means a dot plot that is preceded by a dot plot (Figure 2). This leads to three trial types: pure vs. switch vs. non-switch, and two graph types: bar vs. dot.

Different from the study by Ratwani and Trafton (2008), participants in this study compared the values of two groups. Based on the schematic processes (Kosslyn, 1980; Ullman, 1984; Simkin and Hastie, 1987), one needs the following processes to compare groups. First, *scanning* is necessary to locate the groups of interests and the positions. Second, *projection* is needed to mentally send out a ray from a data point of one group to a data point of the other group. This process can be horizontal or vertical. Third, *comparison* is needed to align the absolute values of the two groups. The positions of the two groups can influence the time needed to make this comparison, as it takes longer to process the relevant information when separated by a larger distance (Moreno and Mayer, 1999; Ayres and Sweller, 2005). Accordingly, one extra factor is considered: the position difference of the group of interests (in our case either 1 vs. 2).

### Research Question

Ratwani and Trafton (2008) used the mixing-costs paradigm and suggested that graph schemas are based on an invariant structure shared by certain categories of graphs. However, their participants had the task to identify a particular value rather than comparing two groups, which would be a task that strongly profits from the power of graphs to support the processing of relational information. Accordingly, this study used a task of group comparison to test the following question and hypotheses.

**Question 1**: Are graph schemas based on perceptual features or common invariant structures shared between certain graph types?

According to the hypothesis of perceptual features, mixing costs should be observed in all pairings of graph types as they all differ in features. According to the hypothesis of invariant structure, only some pairings should produce mixing costs: For instance, tally charts involve a grouping of sub-quantities (quintuples) while in bar graphs and dot plots, quantity is mapped to surface covered without such grouping of subunits. Accordingly, mixing tally charts either with bar graphs or with dot graphs might lead to mixing costs, while mixing bar graphs with dot plots might not lead to mixing costs. The graph schema of tally charts should involve the subgroups of five elements, while the schema used for bar graphs and dot plots does not. This difference in schema should be linked to differences in processing. Tally charts do not contain an axis with a number line, while bar graphs and dot plots offer such a line so that participants can map positions in the graph to numbers on the axis and subtract the numbers.

***Hypothesis 1a***: Bar graph and dot plot share the same graph schema (x-y coordinate), which will lead to similar processing times.

***Hypothesis 1b***: Tally chart does not share the same graph schema with bar graph and dot plot, which will lead to different processing times.

Previous studies showed that bar graphs are more suitable for discrete comparison than line graphs (Zacks and Tversky, 1999; Tversky et al., 2000). However, it is still unclear which type of data graphs can be the best to compare the discrete groups among bar graphs, dot plots, or tally charts. It thus leads to the following question and hypotheses.

**Question 2**: Which graph type (bar, dot, or tally) is the most suitable for group comparison?

***Hypothesis 2a***: Tally chart will have a different processing time than bar graph and dot plot.

***Hypothesis 2b***: The group comparison will be influenced by the position difference of groups of interests. The larger the position difference, the longer the response time will be.

## General Method

### Design and Materials

Three graph types were compared in three experiments (i.e., Experiment 1 on bar vs. dot, Experiment 2 on bar vs. tally, and Experiment 3 on tally vs. dot). Each was a within-subjects design, in which each participant constantly compared the group difference of A and B in different graph types. Each experiment had three blocks (i.e., two pure blocks, one for each graph type, and one mixed block) in 50 trials and in total 150 trials. Each trial consisted of an instruction to compare A and B (the instruction is the same in each trial), a graph, and a text field to type in the answer (Figure 2). Each randomly generated graph displayed the quantities of three groups (A, B, and C). The values ranged from 1 to 9, and the constraint was that the values of the three groups were always different, which led to group differences of A and B between 1 and 8. The three groups had randomized positions from 1 to 3. The position difference of group A and group B could thus be either 1, which means that group A and group B were next to each other, or 2, which means that group A and group B were separated by group C. The sequence of the three blocks was randomized to control for order effects. Participants could take a break after each block. The experiment was programmed in R by using the package Shiny.

Under the mixing-costs paradigm (Ratwani et al., 2008), RTs (i.e., onsets from displaying the graphs to pressing the Enter-key) were compared in two pure blocks (e.g., one block of bar graphs and one block of dot plots) and a mixed block (e.g., one block of both bar graphs and dot plots). The mixed block had four conditions, namely, bar switch, bar non-switch, dot switch, and dot non-switch (Figure 2). There were thus three trial types, namely, pure vs. switch vs. non-switch. Two graphs were examined at a time, in order to determine which graph type is the best to compare the group difference. If a given set of graphs relies on different schemas, it should take more time to load the appropriate schema than if they activate the same schema. A repeated-measures ANOVA was separately performed in each experiment with the following factors: trial type (pure vs. switch vs. non-switch), graph type (e.g., bar vs. dot), and position difference (of A and B: 1 vs. 2). The instructions, experiment program, raw data, and statistical analyses are available online (Zhao, 2021).

### Procedure

Participants were tested in a quiet room and informed about the aim of the study. After signing the declaration of consent, the demographic data of participants and the frequency of using computers were typed in the software. The participants answered ten questions on a 6-point Likert scale regarding their subjective graph literacy (Garcia-Retamero et al., 2016). For instance, “How well can you work with bar graphs?” (1 = *not well at all* to 6 = *extremely well*). Later, the experiment was started in a browser on a Lenovo ThinkPad T530 laptop with a 12.5-inch display. Participants were told to constantly compare the group difference between A and B as accurately and quickly as possible. The participants pressed the keypad numbers to give answers and pressed Enter to go to the next trial. The experiment was part of 5 Bachelor of Science theses, and participants took part voluntarily for no extra reward in the 20-min experiment.

## Experiment 1: Bar vs. Dot

### Participants

An *a priori* power analysis using G^∗^Power 3.1 (Faul et al., 2009) for a repeated-measures ANOVA testing the main effect of trial type (pure vs. switch vs. non-switch) while using two graph types (bar vs. dot) suggested that a sample size of 18 would allow the detection of an effect size of η_*p*_^2^ = 0.10 at α = 0.05 with a statistical power (1–β) = 0.95. Thirty-nine participants (21 females) took part in Experiment 1 (41.3 ± 13.2 years, computer ability with 1 = *never used a computer* to 6 = *everyday use*: 4.3 ± 1.7). The mean age of participants was higher than in many laboratory studies in cognitive psychology, as students of the FernUniversität in Hagen (state-run distance teaching university in Germany) are older and more heterogeneous in age than students at other universities. The graph literacy (Garcia-Retamero et al., 2016) was on average 4.27 ± 0.90 (1 = *not good at all* to 6 = *extremely good*). All participants had normal or corrected-to-normal vision acuity.

### Results

**FIGURE 3:**
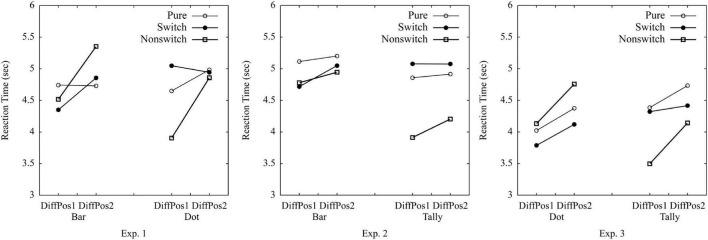
Average reaction times in pure vs. switch vs. non-switch conditions in Experiments 1–3.

The RTs greater than 1 *SD* from the mean among all the participants were excluded (12.920 s, 1.8% of the data). The *SD* cutoff is normally used with a 2 *SD*, 2.5 *SD*, or even a 3 *SD* (cf. Marmolejo-Ramos et al., 2015). This study used a 1-*SD* cutoff, as the data were left-skewed with a large *SD* (7.749 s), and the mean was 5.171 s. If we would have used 2 *SD* (20.669 s) as cutoff, 0.7% of the data would have been excluded. A criterion of 2.5 *SD* (24.543 s) would have resulted in excluding 0.6% of the data; 3 *SD* (28.418 s) would have implied 0.4% of the data being excluded. After excluding the RTs greater than 1 *SD*, the average response time for each trial among all participants was 4.687 s (*SD* = 0.845 s). A repeated-measures ANOVA was conducted on average RTs per participant per condition with the following factors: 3 (trial type: pure vs. switch vs. non-switch) × 2 (graph type: bar vs. dot) × 2 (position difference of A and B: 1 vs. 2) (Table 1 and Figure 3). Crucially, trial type did not reach significance,^1^ *F*(2,76) = 1.12, *p* = 0.33, η_*p*_^2^ = 0.03, suggesting the similar processing time in pure, switch, and non-switch conditions (the estimated Bayes factor was *H*_10_ = 0.68; confirmed ***Hypothesis 1a***). There was no significant effect of graph type either, *F* < 1, indicating similar processing time to compare groups (the estimated Bayes factor was *H*_10_ = 0.13; confirmed ***Hypothesis 2a***). The interaction of trial type × graph type, *F*(1.67,63.63) = 16.48, *p* < 0.001, η_*p*_^2^ = 0.30, indicated that trial type affected the processing times on bar graphs and dot plots differently: Bar graphs were processed quicker with switch and pure trials than non-switch trials, whereas dot plots were processed quicker with non-switch trials and pure trials than switch trials. The effect of position difference was significant, *F*(1,38) = 29.00, *p* < 0.001, η_*p*_^2^ = 0.43, as well as the interactions of trial type × position, *F*(1.36,51.58) = 10.87, *p* < 0.001, η_*p*_^2^ = 0.22, and trial type × graph type × position, *F*(1.55,58.97) = 4.16, *p* = 0.03, η_*p*_^2^ = 0.10, indicating longer RTs with the increase of position difference and the different effect of position on graph type and trial type (confirmed ***Hypothesis 2b***). No other effect was found, graph type × position, *F* < 1. The error rates can be found in the Supplementary Material.

**TABLE 1:** Average reaction times (in seconds) between the position difference of group A and group B (DiffPos) in pure vs. switch vs. non-switch conditions for Experiments 1–3.

	Pure block		Mixed block			
			Switch		Non-switch	
Graph type	DiffPos1	DiffPos2	DiffPos1	DiffPos2	DiffPos1	DiffPos2
**Exp. 1 (*N* = 39)**					
Bar *M*	4.742	4.730	4.351	4.856	4.517	5.354
(*SD*)	(0.884)	(0.942)	(0.861)	(1.222)	(0.814)	(1.872)
Dot	4.650	4.984	5.049	4.944	3.904	4.858
	(1.093)	(1.019)	(1.135)	(1.017)	(0.906)	(1.439)
**Exp. 2 (*N* = 19)**					
Bar	5.115	5.201	4.717	5.049	4.779	4.945
	(1.116)	(1.042)	(1.301)	(1.138)	(0.998)	(1.232)
Tally	4.858	4.915	5.077	5.075	3.913	4.204
	(1.295)	(1.293)	(0.897)	(1.111)	(0.990)	(1.919)
**Exp. 3 (*N* = 40)**					
Dot	4.023	4.375	3.788	4.120	4.132	4.758
	(1.158)	(1.230)	(1.188)	(1.303)	(1.102)	(2.070)
Tally	4.386	4.733	4.322	4.417	3.497	4.143
	(1.262)	(1.280)	(1.097)	(1.147)	(0.987)	(1.575)

### Discussion

There were no mixing costs in Experiment 1. Pure blocks, as well as switch trials and non-switch trials in the mixed block, lead to similar RTs. Therefore, Experiment 1 supports the hypothesis that bar graphs and dot plots share a common invariant structure (Ratwani and Trafton, 2008). Furthermore, bar graphs and dot plots required similar processing times when comparing discrete groups. Apparently, as bar graphs and dot plots both use the x-y coordinate system, no additional graph schema needs to be activated in the mixed block compared to the pure blocks. Comparing groups in bar vs. dot graphs seems to require similar processes. First, one scans for the values of groups A and B by using the x-y coordinate systems. Once it is completed, the difference between A and B is calculated. The position difference of the compared groups plays an essential role. The larger the distance of groups A and B, the longer the RTs.

## Experiment 2: Bar vs. Tally

### Participants

One dataset was excluded due to a technical error, and the data of 19 participants (11 females) were reported in Experiment 2 (36.7 ± 10.5 years, computer ability was 3.5 ± 0.6). The graph literacy was on average 4.36 ± 0.50.

### Results

The RTs greater than 1 *SD* from the mean were excluded (greater than 11.502 s, 3.4% of the data), as the data were left-skewed with a large *SD* of 6.007 s and a mean of 5.494 s. If we would have used 2 *SD* (17.509 s) as cutoff, 1.4% of the data would have been excluded. A criterion of 2.5 *SD* (20.513 s) would have resulted in excluding 1.1% of the data; 3 *SD* (23.517 s) would have implied 0.8% of the data being excluded. After excluding the RTs greater than 1 *SD*, the average response time for each trial among all participants was 4.830 s (*SD* = 1.060 s). Crucially, the 3 (trial type: pure vs. switch vs. non-switch) × 2 (graph type: bar vs. tally) × 2 (position difference of A and B: 1 vs. 2) ANOVAs on average RTs per participant per condition showed the main effect of trial type, *F*(2,36) = 13.28, *p* < 0.001, η_*p*_^2^ = 0.43, and an interaction of trial type × graph type, *F*(2,36) = 7.73, *p* = 0.002, η_*p*_^2^ = 0.30, suggesting that the processing time of bar and tally was different in pure vs. switch vs. non-switch trials (Figure 3). Bar graphs were processed more quickly in switch and non-switch trials than in pure trials, whereas tally charts were more quickly processed in non-switch trials than in pure and switch trials (confirmed ***Hypothesis 1b***). There was a significant effect of graph type, *F*(1,18) = 5.74, *p* = 0.03, η_*p*_^2^ = 0.24, indicating shorter response time on tally charts than on bar graphs (confirmed ***Hypothesis 2a***). The effect of the position reached significance, *F*(1,18) = 5.32, *p* = 0.03, η_*p*_^2^ = 0.23, indicating shorter processing time when the position difference of group A and group B was 1 compared to 2 (confirmed ***Hypothesis 2b***). No other effects were revealed: trial type × position, graph type × position, graph type × trial type × position, *Fs* < 1.

### Discussion

Experiment 2 demonstrated that bar graph and tally chart are not based on the same graph schema. Given the differences (i.e., a grouping of elements of five, axis with numbers), it seems plausible that bar graph and tally chart differ in underlying structure and processes involved. Thus, the mixing costs are consistent with the hypothesis of a common invariant structure (Pinker, 1990; Peebles and Cheng, 2002; Ratwani and Trafton, 2008). Tally charts allowed comparing groups quicker than bar graphs, which might show the advantage of element-based graph schema over x-y coordinates. During the group comparison in a tally chart, one does not need to look at the coordinates to find the values. Furthermore, less time was needed, when the compared groups were near each other.

## Experiment 3: Dot vs. Tally

### Participants

One dataset was excluded due to the missing value in one condition, and the data of 40 participants (17 females) were reported in Experiment 3 (38.5 ± 6.8 years, computer ability: 4.8 ± 1.1). The graph literacy was on average 4.40 ± 0.64.

### Results

The RTs greater than 1 *SD* from the mean were excluded (11.806 s, 2.6% of the data), as the data were left-skewed with a large *SD* of 7.078 s and a mean of 4.728 s. If we would have used a 2 *SD* (18.884 s) as a cutoff, 0.96% of the data would have been excluded. A criterion of 2.5 *SD* (22.423 s) would have resulted in excluding 0.7% of the data; 3 *SD* (25.962 s) would have implied 0.6% of the data being excluded. After excluding the RTs greater than 1 *SD*, the average response time for each trial among all participants was 4.216 s (*SD* = 1.086). Crucially, the 3 (trial type: pure vs. switch vs. non-switch) × 2 (graph type: dot vs. tally) × 2 (position difference of A and B: 1 vs. 2) ANOVAs on average RTs per participant per condition showed a main effect of trial type, *F*(2,78) = 5.12, *p* = 0.008, η_*p*_^2^ = 0.12 (Figure 3), and an interaction of trial type × graph type, *F*(1.48,57.73) = 24.60, *p* < 0.001, η_*p*_^2^ = 0.39, suggesting that the processing time was different in pure vs. switch vs. non-switch trials when processed with dot plots and tally charts (confirmed ***Hypothesis 1b***). In tally charts, non-switch trials were quicker than switch and pure trials. Surprisingly, in dot plots, switch trials were the quickest. There was no significant effect of graph type, *F* < 1, indicating similar response times on tally charts and dot plots (the estimated Bayes factor was *H*_10_ = 0.15; disconfirmed ***Hypothesis 2a***). The effect of position reached significance, *F*(1,39) = 24.49, *p* < 0.001, η_*p*_^2^ = 0.39, as well as the interaction effect of trial type × position, *F*(1.55,60.56) = 4.63, *p* = 0.02, η_*p*_^2^ = 0.11, indicating longer processing times with increased position difference between A and B, and this effect size was different for tally chart and dot plot (confirmed ***Hypothesis 2b***). No other effects were revealed: graph type × position, graph type × trial type × position, *Fs* < 1.

### Discussion

Experiment 3 suggests that tally and dot are not based on the same graph schema. Given the structural differences, this is in line with the hypothesis of the common invariant structure (Ratwani and Trafton, 2008). Surprisingly, in dot plots, switch trials were quicker than pure and non-switch trials. In the mixing-costs paradigm, the mixed block should demand a longer processing time than pure blocks when different graph schemas are activated. One explanation might be that there is a carryover effect in the processing strategy. Participants process tally charts without mapping positions to numbers on the axis (as these elements are not present). In pure blocks with dot plots, participants might often use the axis. Yet, when forced to compare without using the axis in a trial with a tally chart in the mixed block, participants might stick to this quick and direct comparison also in the succeeding dot plot trials. As dot plots (different from bar graphs) do allow to estimate differences directly without first mapping positions to numbers on the axis, switch trials were quicker than non-switch and pure trials in dot plots. The similar processing times of tally charts and dot plots can also be due to the shared feature that both graphs allow direct counting without first mapping with the axis.

## General Discussion

This study tested whether graph schemas are based on perceptual features or common invariant structures. It also examined which graphic type (bar vs. dot vs. tally) is the best to compare discrete groups. There are three main findings.

### Graph Schemas Are Based on a Common Invariant Structure

The mixing-costs paradigm (Los, 1996; Ratwani and Trafton, 2008) was adopted, and the response time was compared in pure blocks and a mixed block (switch vs. non-switch). When two graphs have different graph schemas, the appropriate graph schema should be retrieved in mixed blocks, and this process needs time. When two graphs share the same graph schema, the retrieval process is unnecessary in the mixed block. Consistent with ***Hypotheses 1a and 1b***, bar graphs and dot plots had similar processing times in pure vs. switch vs. non-switch conditions (suggesting a common schema), whereas pairing tally graphs either with dot graphs or with bar graphs led to mixing costs, suggesting that the graph schema of tally charts differs from one of the bar graphs and dot plots. It is possible that bar graphs and dot plots share the common invariant structure of the Cartesian system with the x-y coordinates, and tally charts do not. In the mixed block of bar graphs and dot plots, no extra graph schema needs to be activated. In contrast, in the mixed block of tally chart and bar graph or in the mixed block of the tally chart and dot plot, extra graph schema needs to be activated. The activation of the new graph schema takes time, which leads to the different processing times between tally charts and bar graphs and tally charts and dot plots. The results thus support the hypothesis of a common invariant structure that graph types share current common graph schemas (Pinker, 1990; Peebles and Cheng, 2002, 2003; Ratwani and Trafton, 2008).

### Tally Is Quicker to Compare Discrete Groups Than Bar

Partially in line with ***Hypothesis 2a***, tally charts allow to compare group differences more quickly than bar graphs but have a similar processing time as dot plots. This can be due to the element-based coordinate system and the grouping function (Harris, 1999). Compared to bar graphs, tally charts do not contain the Cartesian coordinate system with x- and y-axis. It consists of an element-based coordinate system, in which all the elements are presented as tally marks. This graph is often used for children to record and count the frequency of data (Nisbet et al., 2003; Ashbrook, 2011; Davis, 2014; Garcia-Retamero et al., 2016). It has a grouping function, i.e., five tally marks create a group with each fifth mark scoring across the previous four marks (Tukey, 1977). This grouping function can be beneficial for group comparisons, as one can divide the comparisons into two fives. For instance, if group A is 3 and group B is 8, the difference is 5. If the data are shown in a tally chart, one can directly count the elements and recognize that the difference is a 5-element unit. If the data are shown in the bar graph, one can only rely on the x-y coordinate system. Similar to tally charts, dot plots contain elements, which are represented as distinct shapes. It is likely that direct group comparison is possible in tally charts and dot plots without first mapping the position of the groups to the axis. As the cognitive processing can overlap, tally charts have a similar processing time of group comparison as the dot plots.

Given the strengths to support comparison, one might ask why tally charts are not a standard graph type in research articles (Borkin et al., 2013). We considered that it might be due to the limitations of the tally chart regarding space and number type. First, research articles normally include a large amount of numbers and have limited space to present the results. The values of A, B, and C are less than 10 in this study. It is unknown whether the tally chart is still the quickest for group comparison when the values of all groups are higher than 10. It is possible that a tally chart is not ideal for the group comparison anymore over a certain number, as one needs much time to count the data. Tukey (1977, S. 16) has mentioned that amounts higher than 20 are difficult to count in tally charts. Future studies should test the maximum value of the tally chart in group comparison. In addition, the tally chart can present integers but cannot represent floating numbers or percentages very well (e.g., 3.5 or 14%). In this study, the values of all groups are only integers instead of numbers with decimals. Therefore, space and number type limit the use of tally chart in a broader aspect, such as in research articles.

### Smaller Position Difference Between Compared Groups Leads to the Shorter Processing Time

In all three experiments, we observed that group comparisons were quick when the groups of interests were next to each other, which confirmed ***Hypothesis 2b***. Eye-tracking studies (Moreno and Mayer, 1999; Johnson and Mayer, 2012) have shown that longer saccades are needed when the relevant information is presented at a larger distance. A study on text reading (Bower et al., 1979) also suggests that reading is faster when the statement is at a 1-step distance compared to larger distances (i.e., 2-step and 3-step distance). When comparing the values of the two groups, shorter distance demands shorter saccades, which can lead to shorter response time. The results also provide empirical implications: One should set the groups of interests near each other to save time during comparison.

### Limitations

In tally charts, five numbers are grouped into one unit, which can be beneficial for group comparison. The grouping function can be added by using grid lines in the bar and dot graphs (Culbertson and Powers, 1959; Goncu et al., 2010). It is suggested that grid-on-graph and grid-underlay-graph are more efficient (more accuracy and less time) to process than no-grid graphs (Lederman and Campbell, 1983). Participants had higher accuracy with grid graphs than with no-grid graphs (Aldrich and Parkin, 1987). Further studies should test whether the grid lines can assist the group comparison in graphs. This study uses the task of comparing the absolute value of two groups. Follettie (1986) has reported that there is a difference between measurement by reporting the absolute values of two groups, discrimination by responding with the higher value of a group, and comparative estimation by giving the relative values of two groups. Further studies should test the difference between absolute values, higher values, and relative values. Moreover, this study used horizontal bar graphs, and a previous study showed that horizontal bars are slightly favored over vertical bars (Culbertson and Powers, 1959). Further studies should compare the horizontal and vertical bars. Lastly, there are several types of dot plots, such as to show only the dots at the highest value (Sönning, 2018) or to show big dots at the highest value and small dots in between (Cleveland and McGill, 1985). It might be worthwhile to examine whether these types of dot plots share the same graph schema compared to bar graphs.

Data graphs can support the comparison of conditions that represent the results of different types of research designs. In this study, we have designed stimuli in line with a one-factorial design as the simplest possible case in mind. Yet, future work should extend the comparison of counts to two-factorial designs. Using stimuli representing continuous values, a previous study (Ali and Peebles, 2013) has examined how to improve the accuracy with which participants correctly interpret the plots of two-way ANOVA designs (with respect to the question of whether there is an interaction or not). Different from this study, the plots were not about discrete numbers nor was identifying a quantity relevant. Yet, the authors identified how the graph layout can be set up that attention is automatically allocated to aspects that are otherwise often ignored, which leads to misinterpretations. In particular, attention was (by color mappings or by using bar graphs) directed to the x-axis (which according to verbal protocols was otherwise often ignored). Future studies should thus take plots for 2 × 2 designs into account. In relation to the two-way ANOVA designs with count data, other visual techniques (e.g., mosaic plot in Theus, 2012) should be considered in future studies. Moreover, bar graphs are not optimal if the average should be estimated, such as the distribution of data (Newman and Scholl, 2012; Jambor, 2016). Future studies should also consider continuous data in the two-way ANOVA design with other visual techniques, such as boxplots (Hubert and Vandervieren, 2008), violin plots (Thrun et al., 2020), to deliver a clear picture of Empirical Cumulative Distribution Functions (ECDFs) and Probability Density Function (PDFs).

## Conclusion

Dot plots (a variant of bar graphs) and tally charts (often used by children) have been rarely focused on in the research of graph comprehension. This study sheds light on understanding the graph schema underlying these graph types compared to bar graphs. This study supports the invariant structure view (Peebles and Cheng, 2002; Ratwani and Trafton, 2008) that dot plots, which is a variant of bar graphs, share the same graph schema as bar graphs. Tally charts do not consist of x-y coordinates and do not share the same graph schema with bar and dot graphs. The search path of group comparison in tally charts can be shorter than in bar graphs. This study also implies that groups of interests should be presented next to each other to reduce the comparison time.

## Data Availability Statement

The original contributions presented in the study are included in the article/Supplementary Material, further inquiries can be directed to the corresponding author.

## Ethics Statement

The studies involving human participants were reviewed and approved by the Ethics Committee of the German Psychological Association (April 19, 2018). The patients/participants provided their written informed consent to participate in this study.

## Author Contributions

FZ conceptualized the study, wrote the program, organized the data collection, analyzed the data, and prepared the manuscript. Both authors jointly edited the manuscript and approved it for submission.

## Conflict of Interest

The authors declare that the research was conducted in the absence of any commercial or financial relationships that could be construed as a potential conflict of interest.

## Publisher’s Note

All claims expressed in this article are solely those of the authors and do not necessarily represent those of their affiliated organizations, or those of the publisher, the editors and the reviewers. Any product that may be evaluated in this article, or claim that may be made by its manufacturer, is not guaranteed or endorsed by the publisher.

## Footnotes

1 The Friedman’s test for non-parametric dataset was conducted in three experiments to examine the robustness of the results. They were fully consistent with the pattern of the parametric analyses. There was no significant effect of trial type (pure vs. switch vs. non-switch) in Experiment 1 (Bar vs. Dot), χ^2^_*F*_(2) = 2.67, *p* = 0.26. In Experiment 2 (Bar vs. Tally), there was a significant effect of trial type, χ^2^_*F*_(2) = 11.37, *p* = 0.003. Also, in Experiment 3 (Tally vs. Dot), there was a significant effect of trial type, χ^2^_*F*_(2) = 15.80, *p* < 0.001. The analyses of using medians per cell can be found in the Supplementary Material. The median-based analysis showed a significant main effect of trial type only in Experiment 2.
