# Supplementary material for: Best Graph Type to Compare Discrete Groups: Bar, Dot, and Tally
Source: Front Psychol. 2021 Dec 24;12:775721. doi: 10.3389/fpsyg.2021.775721 (PMC8740269; doi:10.3389/fpsyg.2021.775721)

Supplementary Material

# Error rates

**Table 1A**

Error rates (in %) between difference of positions of Group A and Group B (DiffPos) in pure trials and switch and nonswitch trials for Experiments 1-3

|  | Pure  Block | | Mixed Block | | | |
| --- | --- | --- | --- | --- | --- | --- |
|  |  |  | Switch | | Nonswitch | |
| Graph Type | DiffPos1 | DiffPos2 | DiffPos1 | DiffPos2 | DiffPos1 | DiffPos2 |
| Exp. 1 (N = 39) |  |  |  |  |  |  |
| Bar *M(SD)* | 14.6% | 8.1% | 8.1% | 10.1% | 7.2% | 5.1% |
|  | (12.9%) | (10.8%) | (12.8%) | (19.7%) | (11.7%) | (22.3%) |
| Dot | 9.3% | 8.2% | 8.2% | 5.9% | 5.9% | 1.3% |
|  | (10.3%) | (8.2%) | (18.2%) | (8.9%) | (9.7%) | (8.0%) |
| Exp. 2 (N = 19) |  |  |  |  |  |  |
| Bar | 7.5% | 4.7% | 2.5% | 3.2% | 3.5% | 0.0% |
|  | (6.9%) | (6.4%) | (6.0%) | (7.5%) | (8.5%) | (0.0%) |
| Tally | 3.5% | 5.5% | 4.2% | 5.3% | 3.2% | 0.0% |
|  | (4.3%) | (3.9%) | (8.4%) | (8.0%) | (3.9%) | (0.0%) |
| Exp. 3 (N = 41) |  |  |  |  |  |  |
| Dot | 6.7% | 2.6% | 1.6% | 2.0% | 3.3% | 2.5% |
|  | (5.5%) | (3.8%) | (5.0%) | (6.1%) | (6.5%) | (15.8%) |
| Tally | 4.2% | 5.8% | 5.0% | 7.2% | 1.9% | 0.0% |
|  | (4.6%) | (5.6%) | (10.1%) | (10.7%) | (3.8%) | (0.0%) |

**Experiment 1: Bar vs. Dot.** The mean error rate for each trial among all participants was 9.46% (*SD* = 8.66%). The corresponding ANOVA on error rates revealed a main effect of trial type, *F*(1.63, 61.75) = 9.41, *p* = .001, η_p_^2^ = .20, indicating higher error rates in pure trials than in switch and nonswitch trials [here and elsewhere the greenhouse-Geisser correction was applied](see Figure 1A). There was no significant effect of graph type, *F*(1, 38) = 3.28, *p* = .08, η_p_^2^ = .08, suggesting similar accuracy of group comparisons for bar graphs and dot plots. There was a marginal effect of position, *F*(1, 38) = 4.07, *p* = .051, η_p_^2^ = .10, suggesting that the larger the position difference, the lower the error rates are. No other effect was found, trial type × graph type, *F* < 1; trial type × position, *F*(2, 76) = 1.62, *p* = .21, η_p_^2^ = .04; graph type × position, *F* < 1; graph type × trial type × position, *F*(2, 76) = 2.01, *p* = .14, η_p_^2^ = .05.

**Experiment 2: Bar vs. Tally.** The mean error rate among all participants was 4.7% (*SD* = 3.3%). The corresponding ANOVA on error rates revealed a main effect of trial type, *F*(2, 36) = 7.65, *p* = .002, η_p_^2^ = .30, indicating different effects of trial type (see Figure 1A). There was no significant effect of graph type, *F* < 1. No other effect was found, position, *F*(1, 18) = 3.17, *p* = .09, η_p_^2^ = .15; trial type × graph type, *F*(1.28, 23.01) = 1.79, *p* = .20, η_p_^2^ = .09, trial type × position, *F*(2, 36) = 2.86, *p* = .07, η_p_^2^ = .14, graph type × position, *F*(1, 18) = 2.19, *p* = .16, η_p_^2^ = .11, graph type × trial type × position, *F*(1.50, 26.93) = 1.33, *p* = .28, η_p_^2^ = .07.

**Experiment 3: Dot vs. Tally.** The mean error rate for each trial among all participants was 4.4% (*SD* = 2.6%). The corresponding ANOVA on error rates revealed a main effect of trial type, *F*(2, 78) = 3.95, *p* = .02, η_p_^2^ = .10, and an interaction effect of trial type × graph type, *F*(2, 78) = 7.49, *p* = .001, η_p_^2^ = .16, indicating different effects of trial type in dot graphs compared to tally charts: Dot graphs had higher error rates in pure trials than in switch and nonswitch trials, whereas tally charts had higher error rates in switch trials than in pure and nonswitch trials (see Figure 1A). The main effect of graph type, *F*(1, 39) = 4.61, *p* = .04, η_p_^2^ = .11, and an interaction effect of graph type × position, *F*(1, 39) = 7.65, *p* = .01, η_p_^2^ = .16, indicating lower error rate with dot plots than tally charts and the effect of graph type was at different size on different positions. No other effect was found, position, *F* < 1, trial type × position, *F* < 1, graph type × trial type × position, *F*(1.63, 63.47) = 1.06, *p* = .34, η_p_^2^ = .03.


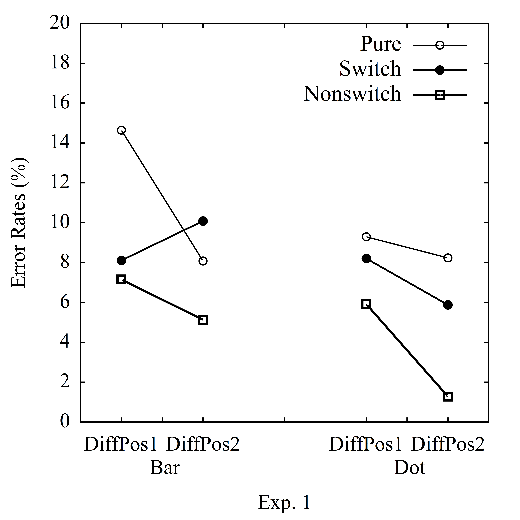

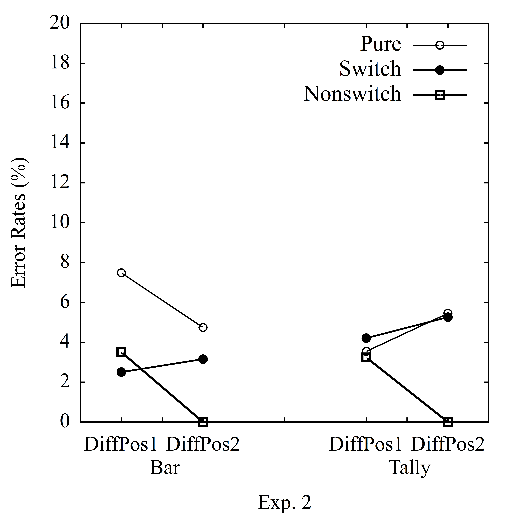


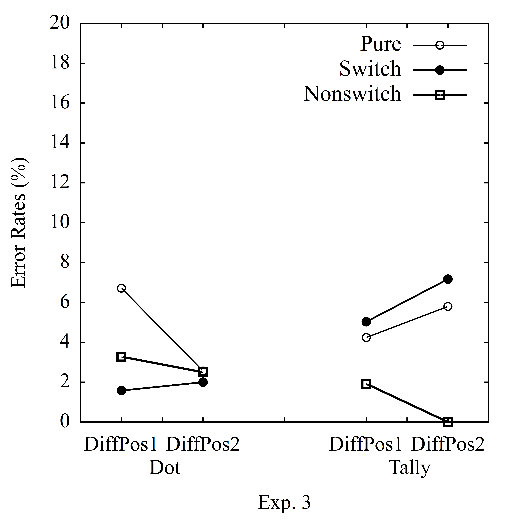


**Supplementary Figure 1A.** Average error rates in pure vs. switch vs. nonswitch conditions in Experiment 1-3.

**2. Analyses using median**

**Experiment 1: Bar vs. Dot.** The average median of RTs among all participants was 4.413 sec (*SD* = 0.828 sec). A repeated-measures ANOVA was conducted on average median of RTs per participant per condition with the following factors: 3 (trial type: pure vs. switch vs. nonswitch) × 2 (graph type: bar vs. dot) × 2 (position difference of A and B: 1 vs. 2). There was no significant effect of trial type, *F*(2, 76) = 1.17, *p* = .32, η_p_^2^ = .03, indicating similar processing time in pure, switch and nonswitch trials, and an interaction effect of trial type × graph type, *F*(2, 76) = 16.31, *p* < .001, η_p_^2^ = .30, indicating the effect of trial type was greater in dot plots than in bar graphs (see Figure 2A). The corresponding ANOVA on average median did not reveal any significant effect of graph type, *F* < 1, suggesting similar processing time of group comparisons for bar graphs and dot plots. There was a main effect of position, *F*(1, 38) = 39.98, *p* < .001, η_p_^2^ = .51, suggesting that the larger the position difference, the longer was the processing time. An interaction effect of trial type × position, *F*(1.33, 50.33) = 20.01, *p* < .001, η_p_^2^ = .35, and an interaction effect of graph type × trial type × position, *F*(1.49, 56.59) = 4.55, *p* = .02, η_p_^2^ = .11, suggested that this effect was larger for switch and nonswitch trials than pure trials for bar plots, whereas this effect was larger in pure and nonswitch trials than in switch trials for dot plots. No other effect was found, graph type × position, *F* < 1.

**Experiment 2: Bar vs. Tally.** The average median of RTs among all participants was 4.669 (*SD* = 1.095). The corresponding ANOVA on average median of RTs revealed a main effect of trial type, *F*(2, 38) = 11.00, *p* < .001, η_p_^2^ = .38, and an interaction effect of trial type × graph type, *F*(1.51, 27.17) = 9.90, *p* = .001, η_p_^2^ = .36, indicating different effects of trial type in bar graphs compared to tally charts: Bar graphs had longer processing time in pure trials than in switch and nonswitch trials, whereas tally charts had longer processing time in pure and switch trials than in nonswitch trials (see Figure 2A). There was no significant effect of graph type, *F*(1, 18) = 3.04, *p* = .10, η_p_^2^ = .14, indicating similar processing time of group comparisons for bar graph and tally charts. There was a main effect of position, *F*(1, 18) = 4.51, *p* = .048, η_p_^2^ = .20, suggesting that the larger the position difference, the longer was the processing time. No other effect was found, trial type × position, *F*(1.31, 23.60) = 1.77, *p* = .20, η_p_^2^ = .09, graph type × position, *F* < 1, graph type × trial type × position, *F*(1.45, 26.14) = 2.25, *p* = .14, η_p_^2^ = .11.

**Experiment 3: Dot vs. Tally.** The average median of RTs among all participants was 3.956 sec (*SD* = 1.191 sec). The corresponding ANOVA on average median of RTs did not reveal an effect of trial type, *F*(2, 78) = 1.14, *p* = .33, η_p_^2^ = .03, but an interaction effect of trial type × graph type, *F*(1.71, 66.77) = 16.27, *p* < .001, η_p_^2^ = .29, indicating different effects of trial type in dot graphs compared to tally charts: Dot graphs had shorter processing time in switch trials than in nonswitch trials and pure trials, whereas tally charts had shorter processing time in nonswitch trials than in pure and switch trials (see Figure 2A). There was no significant effect of graph type, *F*(1, 39) = 1.88, *p* = .18, η_p_^2^ = .05, indicating similar processing time of group comparisons for dot graph and tally charts. There was a main effect of position, *F*(1, 39) = 27.67, *p* < .001, η_p_^2^ = .42, suggesting that the larger the position difference, the longer was the processing time. An interaction effect of trial type × position, *F*(1.58, 61.57) = 5.80, *p* = .01, η_p_^2^ = .13, and an interaction effect of graph type × trial type × position, *F*(1.68, 65.54) = 5.05, *p* = .01, η_p_^2^ = .12, indicating the effect of position was larger in nonswitch trials compared to switch and pure trials for dot plots, whereas the effect of position was larger in nonswitch and pure trials compared to switch trials for tally charts. No other effect was found, graph type × position, *F* < 1.

**Figure 2A**

Average reaction times (in seconds) by using the average median of RTs per participant per condition between position difference of group A and group B (DiffPos) in pure vs. switch vs. nonswitch conditions for Experiments 1-3


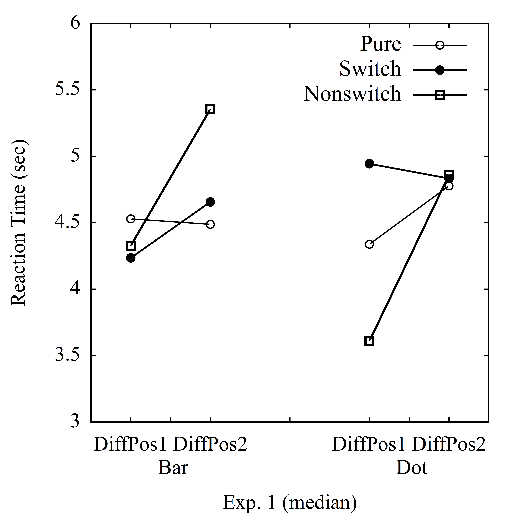

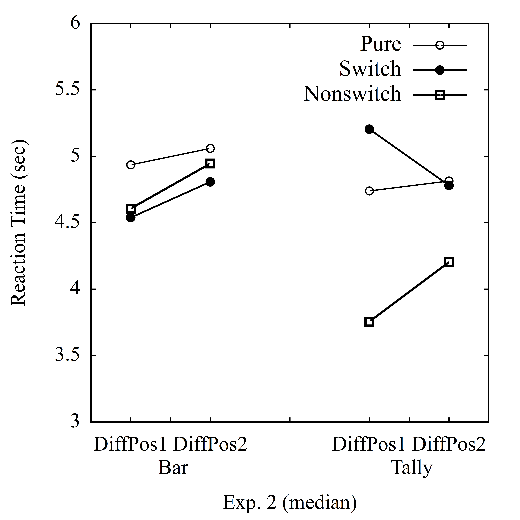


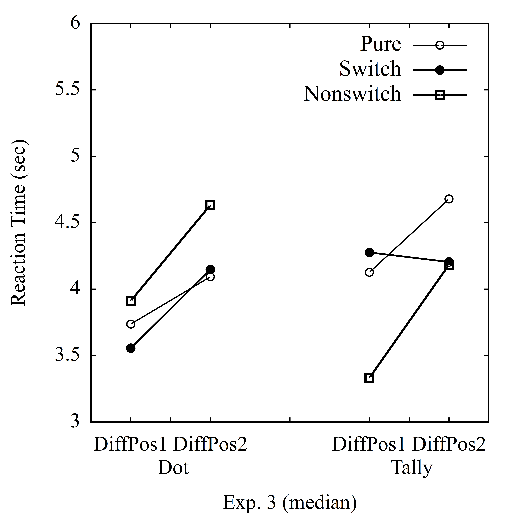

Supplement: Supplementary file 1 [file Data_Sheet_1.docx]
